# A COMMUNITY-ENGAGED APPROACH TO CULTURAL ADAPTATION OF BEHAVIORAL INTERVENTIONS: ENGAGING LATINOS IN RESEARCH

**DOI:** 10.1093/geroni/igae098.1169

**Published:** 2024-12-31

**Authors:** Maria Quiñones-Cordero, Silvia Sörensen, Kenneth Hepburn, Kathi Heffner

**Affiliations:** University of Rochester Medical Center, Rochester, New York, United States; University of Rochester, Rochester, New York, United States; Emory University, Atlanta, Georgia, United States; University of Rochester, Rochester, New York, United States

## Abstract

Latinos are 1.5 times more likely to develop Alzheimer’s disease and related dementias (ADRD) than non-Hispanic white Americans. Latinos also have the highest prevalence of caring for a loved one with ADRD. They spend more time in caregiving-related activities, have limited social support and access to quality dementia care, and have significantly worse physical and mental health. The paucity of culturally congruent interventions to improve Latino ADRD caregivers’ psychological well-being continues to perpetuate poorer outcomes and disparities among Latino caregivers. While several effective psychoeducation and skill-building caregiver programs have been developed, these interventions lack representation of Latino caregivers. Thus, culturally attuned adaptations and implementation of available interventions for Latinos are needed to address this critical gap. This study engaged community-dwelling older Latinos, including Latino caregivers, and Latino community organization leaders, to collaborate in cultural adaptation of the Tele-Savvy program and clinical trial design. Employing community-engaged approaches, we combined a staged cultural adaptation framework and a locally developed training program to engage Latinos in caregiving research. Nine Latino community members served on a community advisory board (CAB). Members first engaged in a training program to develop knowledge about ADRD and skills in conducting research and cultural adaptation. Following the training, members engaged in cultural adaptation of Tele-Savvy and the design of a future clinical trial to test the adapted intervention with Latino ADRD caregivers. We will discuss preliminary findings from the cultural adaptation work with CAB members and future directions to inform the testing of the adapted Tele-Savvy and its refinement.

